# Genetic Interaction Landscape Reveals Critical Requirements for *Schizosaccharomyces pombe* Brc1 in DNA Damage Response Mutants

**DOI:** 10.1534/g3.115.017251

**Published:** 2015-03-19

**Authors:** Arancha Sánchez, Assen Roguev, Nevan J. Krogan, Paul Russell

**Affiliations:** * Department of Cell and Molecular Biology, The Scripps Research Institute, La Jolla, California 92037; † Department of Cellular and Molecular Pharmacology, University of California, San Francisco, California 94158

**Keywords:** *Schizosaccharomyces pombe*, Brc1, CSN/signalosome complex, DNA damage response

## Abstract

Brc1, which was first identified as a high-copy, allele-specific suppressor of a mutation impairing the Smc5-Smc6 holocomplex in *Schizosaccharomyces pombe*, protects genome integrity during normal DNA replication and when cells are exposed to toxic compounds that stall or collapse replication forks. The C-terminal tandem BRCT (BRCA1 C-terminus) domain of fission yeast Brc1 docks with phosphorylated histone H2A (γH2A)-marked chromatin formed by ATR/Rad3 checkpoint kinase at arrested and damaged replication forks; however, how Brc1 functions in relation to other genome protection modules remains unclear. Here, an epistatic mini-array profile reveals critical requirements for Brc1 in mutants that are defective in multiple DNA damage response pathways, including checkpoint signaling by Rad3-Rad26/ATR-ATRIP kinase, DNA repair by Smc5-Smc6 holocomplex, replication fork stabilization by Mrc1/claspin and Swi1-Swi3/Timeless-Tipin, and control of ubiquitin-regulated proteolysis by the COP9 signalosome (CSN). Exogenous genotoxins enhance these negative genetic interactions. Rad52 and RPA foci are increased in CSN-defective cells, and loss of γH2A increases genotoxin sensitivity, indicating a critical role for the γH2A-Brc1 module in stabilizing replication forks in CSN-defective cells. A negative genetic interaction with the Nse6 subunit of Smc5-Smc6 holocomplex indicates that the DNA repair functions of Brc1 and Smc5-Smc6 holocomplex are at least partially independent. Rtt107, the Brc1 homolog in *Saccharomyces cerevisiae*, has a very different pattern of genetic interactions, indicating evolutionary divergence of functions and DNA damage responses.

Genome stability is especially at risk during the DNA synthesis (S) phase of the cell cycle, when relatively innocuous DNA lesions can impede replication or be converted into dangerous chromosome breaks by passage of the replisome. These DNA lesions may originate from DNA replication errors, from toxic endogenous molecules such as free radicals arising from normal cellular metabolism, or from a wide variety of exogenous sources. Ancient prokaryotes evolved many of the most critical mechanisms for protecting genome integrity, such as homology directed repair, base excision repair, and mismatch repair. Eukaryotes inherited these mechanisms and added many more, such that even single-cell eukaryotes possess a complex array of genome protection pathways.

Brc1 protein in *Schizosaccharomyces pombe* plays an important role in maintaining genome stability and yet its mechanism of action remains poorly understood. Brc1 was first identified as an allele-specific, high-copy suppressor of *smc6-74*, which impairs the function of the Smc5-Smc6 holocomplex (Verkade *et al.* 1999). As with other members of the evolutionarily conserved SMC (structural maintenance of chromosomes) family of proteins, the Smc5−Smc6 complex is critical for chromosome segregation and is also important for DNA repair, especially at collapsed replication forks (De Piccoli *et al.* 2009; Kegel and Sjogren 2010; Pebernard *et al.* 2006). Brc1 is not essential for cell viability, but it is required in strains with compromised functions of the Smc5−Smc6 complex (Morikawa *et al.* 2004; Pebernard *et al.* 2004; Verkade *et al.* 1999). Brc1-defective strains are sensitive to genotoxins that stall replication forks or create DNA lesions that lead to replication fork collapse or other forms of replication stress (Sheedy *et al.* 2005). Furthermore, *brc1Δ* cells have increased Rad52 foci, which indicate DNA replication difficulties even in the absence of exogenous genotoxins (Bass *et al.* 2012; Williams *et al.* 2010).

The presence of six BRCT (BRCA1 carboxyl terminus) domains is a defining structural feature of Brc1 that is shared with the evolutionary conserved *Saccharomyces cerevisiae* Rtt107 and human PTIP proteins (Munoz *et al.* 2007; Rouse 2004). These proteins also share the ability to bind histone H2A (or H2AX in mammals) that has been phosphorylated the ATM/ATR family of master DNA damage response checkpoint kinases (Li *et al.* 2012; Manke *et al.* 2003; Williams *et al.* 2010; Yan *et al.* 2011). This chromatin-specific interaction is mediated through the C-terminal pair of BRCT domains as also seen in DNA damage response mediator proteins such as human Mdc1 and fission yeast Crb2 (Du *et al.* 2006; Kilkenny *et al.* 2008; Stucki *et al.* 2005). Despite the overall structural similarities of Brc1, Rtt107, and PTIP and their importance for protecting genome integrity, it remains unclear whether they have conserved functions. Here, we investigate Brc1 by generating an epistatic miniarray profile (E-MAP) consisting of the quantitative analysis of genetic interactions between *brc1Δ* and a *S. pombe* gene deletion library (Roguev *et al.* 2007). These E-MAP data provide novel insights into the functional relationships between Brc1 and other genome protection pathways in fission yeast.

## Materials and Methods

### Strains and genetic methods

The strains used in this study are listed in Supporting Information, Table S1. Standard fission yeast methods were used as described previously (Forsburg and Rhind 2006). New null alleles of *csn1*, *csn5*, *ddb1*, *spd1*, *pnk1*, *sde2*, *raf1*, and *snt1* were constructed using targeting constructs in which the entire open reading frames were replaced by *KanMX6* as described previously (Bahler *et al.* 1998). Successful deletion of these genes was verified by polymerase chain reaction. Tetrad analysis was performed to construct double mutants and verified by polymerase chain reaction.

### Epistatic miniarray profile (E-MAP)

E-MAP screens were performed and normalized as described previously (Roguev *et al.* 2008). Complete E-MAP profiles can be found in File S1.

### Gene Ontology (GO) analysis

GO enrichment analysis used the Princeton implementation of GO term finder (http://go.princeton.edu/cgi-bin/GOTermFinder) (Boyle *et al.* 2004). Analysis used a p-value cut off of 0.01. For the fission yeast *brc1Δ* E-MAP, the 56 SSL genes were compared with the background population of 2026 genes that produced E-MAP values (File S2). For the budding yeast *rtt107Δ* E-MAP, the 33 SSL genes (Collins *et al.* 2007) were compared with a background population consisting of all genes in budding yeast (File S3).

### Survival assay

DNA damage sensitivity assays were performed by spotting 10-fold serial dilutions of exponentially growing cells onto yeast extract with glucose and supplements plates, and treated with indicated amounts of hydroxyurea (HU), camptothecin (CPT), and methyl methanesulfonate (MMS). For ultraviolet (UV) treatment, cells were serial diluted onto yeast extract with glucose and supplements plates and irradiated using a Stratagene Stratalinker UV source. Cell survival was determined after 3-4 d at 30°.

### Microscopy

Cells were photographed using a Nikon Eclipse E800 microscope equipped with a Photometrics Quantix charge-coupled device camera and IPlab Spectrum software. All fusion proteins were expressed at their own genomic locus. Rad52-yellow fluorescent protein (YFP)− and RPA (Rad1)-green fluorescence protein−expressing strains were grown in Edinburgh minimal medium until mid-log phase for focus quantification assays. Quantification was performed by scoring 500 or more nuclei from three independent experiments.

## Results

### Quantitative genetic interaction analysis of Brc1

To gain new functional insights into Brc1 we carried out an E-MAP analysis to quantify the genetic interactions between *brc1Δ* and a *S. pombe* gene deletion library of nonessential genes (Kim *et al.* 2010; Roguev *et al.* 2007). E-MAP values were determined with a simple growth phenotype that measures negative (aggravating) interactions, such as synthetic sick/lethal (SSL) interactions, as well as positive (alleviating) interactions in which the double mutant is healthier than would be expected based on the growth of the two single mutants. An SSL interaction often identifies proteins that function in distinct but parallel pathways, whereas a positive interaction score may indicate either suppression or masking effects, in which loss of one gene masks the effect of losing another, as seen when two proteins act together in a common complex or pathway (Collins *et al.* 2007; Roguev *et al.* 2007).

The resulting Brc1 E-MAP consists of 2026 interaction scores (Table S2). Of these, 56 genes displayed a significant negative genetic interaction with *brc1∆* (interaction score < −2.5) and 23 displayed positive genetic interactions (interaction score >2). Most genes have genetic interactions scores close to zero. The results of this screen are summarized in Table S2.

GO analysis of the SSL mutants identified in this screen revealed significant enrichment of genes involved in key cellular processes, including cellular response to DNA damage stimulus, DNA repair, DNA damage checkpoint, chromatin modification, and cullin deneddylation (Table 1). The strongest SSL score was obtained with *csn1* (SSL = −15.1), which encodes a subunit of the COP9/Signalosome (CSN) complex that has important functions in the protection of genome integrity (Mundt *et al.* 1999). Genome protection was also highlighted by other genes with the greatest SSL scores, such as *apn2* (SSL = −14.6), which encodes an apurinic/apyrimidinic endonuclease required for base excision repair (Fraser *et al.* 2003), *hrq1* (SSL = −14.1), which encodes a RecQ type DNA helicase that plays an important role in DNA interstrand cross-link repair (Groocock *et al.* 2012), and *rad26* (SSL = −12.5), which encodes an ATRIP ortholog required for the activity of Rad3/ATR checkpoint kinase (Edwards *et al.* 1999). For comparison, the recently analyzed *brc1* SSL interaction with *dcd1*, which encodes a deoxycytidylate deaminase required to maintain a proper balance of dNTPs, was −7.8 in this screen (Sanchez *et al.* 2012). All of these data are consistent with Brc1 playing an important role in genome protection. GO analysis of the 23 genes that displayed positive genetic interactions with *brc1Δ* failed to yield specific process enrichment terms.

**Table 1:** Summary of significant enriched GO categories for biological function of genes with genetic interactions positively correlated with *brc1* (*P* ≤ 0.01)

Process	Brc1 E-MAP Functional Groups
Cellular response to DNA damage stimulus	srs2, csn1, rad1, dbl1, rad2, ssb3, rad9, rad17, pku80, hus1, hat1, ddb1, mms22, rad26, hrq1, nse6, arp42, apn2, pnk1, swi3
DNA repair	srs2, ddb1, mms22, rad1, rad2, ssb3, rad9, rad17, pku80, hus1, hrq1, arp42, pnk1, apn2, hat1
DNA damage checkpoint	hus1, rad26, rad1, dbl1, rad9, rad17
Chromatin modification	ubp8, ngg1, spt3, pmt3, nrl1, clr1, raf1, arp42, set1, snt1, hat1
Cullin deneddylation	csn1, csn5, csn71
Others	cbf11, dcd1, vps60, SPBC1711.15C, SPBC1289.14, mpp6, SPAC1635.01, SPAC1071.09C, SPBP16F5.05C, naa30, nup60, SPBP16F5.08C, rga8, SPBC651.02, urm1,SPCC1442.02, ppk3, sde2, SPBC27.05, pmp3, SPBC1711.09C, hmt1, atg22, fep1

GO, Gene Ontology; E-MAP, epistatic miniarray profile.

### Comparison of Brc1 and Rtt107 E-MAPs

Fission yeast Brc1 and budding yeast Rtt107 are 6-BRCT domain proteins that bind γH2A and are important for survival of DNA lesions formed in S-phase (Bogliolo *et al.* 2007; Cobb *et al.* 2005; Fernandez-Capetillo *et al.* 2004; Marti *et al.* 2006; Papamichos-Chronakis and Peterson 2008; Ward and Chen 2001), yet it remains unclear whether they are functional orthologs. It was therefore of interest to compare the SSL E-MAP data for Brc1 and Rtt107 (Beltrao *et al.* 2010; Collins *et al.* 2007). We found that of the 56 *S. pombe* genes and 33 *S. cerevisiae* genes (Table S3 and Table S4), the E-MAP overlap encompassed only one gene, *srs2/SRS2*, which is an ATP-dependent DNA helicase that functions as an anti-recombinase but is also required for efficient repair of DSBs in S-phase (Doe and Whitby 2004; Liu *et al.* 2011) This small degree of gene-for-gene overlap was surprising in view of the overall structural similarity of Brc1 and Rtt107. Moreover, global E-MAP comparisons of *S. pombe* and *S. cerevisiae* detected significant conservation of negative genetic interactions between genes with the same functional annotation (Roguev *et al.* 2008). Evolutionary divergences in key genome maintenance pathways likely account for some of E-MAP differences between Brc1 and Rtt107. For example, our Brc1 E-MAP includes subunits of the CSN protein complex (Mundt *et al.* 1999), which is absent in *S. cerevisiae*. Indeed, when we compared GO process analysis of negative genetic interactions of Brc1 (Table 1 and File S2) and Rtt107 E-MAPs (Table 2 and File S3), both highlighted significant enrichment in interactions with genes involved in DNA damage stimulus and DNA repair. On the other hand, other key GO process terms that were highly enriched in the Brc1 E-MAP analyses were absent in the comparable Rtt107 lists, notably DNA damage checkpoint and chromatin modification. Likewise, key GO process terms enriched for genes having negative genetic interactions with Rtt107 were absent the Brc1 E-MAP list, including nuclear division and double-strand break repair. Taken as a whole, these data suggest that although *S. pombe* Brc1 and *S. cerevisiae* Rtt107 are both involved in protecting genome integrity in response to DNA lesions that arise or are repaired preferentially in S-phase, their patterns of genetic interactions are not highly conserved, which indicates either substantial functional differences between Brc1 and Rtt107, large differences between *S. pombe* and *S. cerevisiae* in the mechanisms that maintain genome integrity or cause replicative stress when defective, or a combination of these effects.

**Table 2:** Summary of significant enriched GO categories for biological function of genes with genetic interactions positively correlated with RTT107 (*P* ≤ 0.01)

Process	RTT107 E-MAP Functional Groups
DNA metabolic process	RRM3, MND2, SGS1, RMI1, POL30, SPT4, TSA1, MRE11, SWI6, XRS2, RTT101, SLX5, TEL1, TOP3, SRS2, MSH1, NEJ1, NUP84
DNA repair	RRM3, MRE11, XRS2, SGS1, SLX5, TEL1, SRS2, MSH1, NEJ1, POL30, NUP84, SPT4
Double-strand break repair	SRS2, TEL1, NEJ1, NUP84, MRE11, XRS2, SGS1
Double-strand break repair via nonhomologous end joining	SRS2, NEJ1, MRE11, XRS2
Response to stress	RRM3, YOR338W, SGS1, RMI1, POL30, SPT4, TSA1, SWI6, XRS2, MRE11, RTT101, SLX5, TEL1, SRS2, MSH1, NEJ1, NUP84
Telomere maintenance and organization	TOP3, TEL1, RRM3, XRS2, SGS1, SLX5
Cell cycle	CLB2, TSA1, MND2, MRE11, XRS2, SWI6, YOR338W, SGS1, RTT101, RMI1, TEL1, TOP3, CDC10, POL30
Chromosome organization	RRM3, MND2, XRS2, SGS1, SLX5, RMI1, TEL1, TOP3, POL30, NUP84, SPT4
DNA recombination	SRS2, TOP3, MND2, MRE11, SWI6, SGS1
Others	RPN6, RPA190, YNR048W, GET2, AIM4, REB1, NUT1, BEM2, RPN10, TAH1, SRP40, DST1

GO, Gene Ontology; E-MAP, epistatic miniarray profile.

### Genotoxins enhance Brc1 genetic interactions

**Figure 1:**
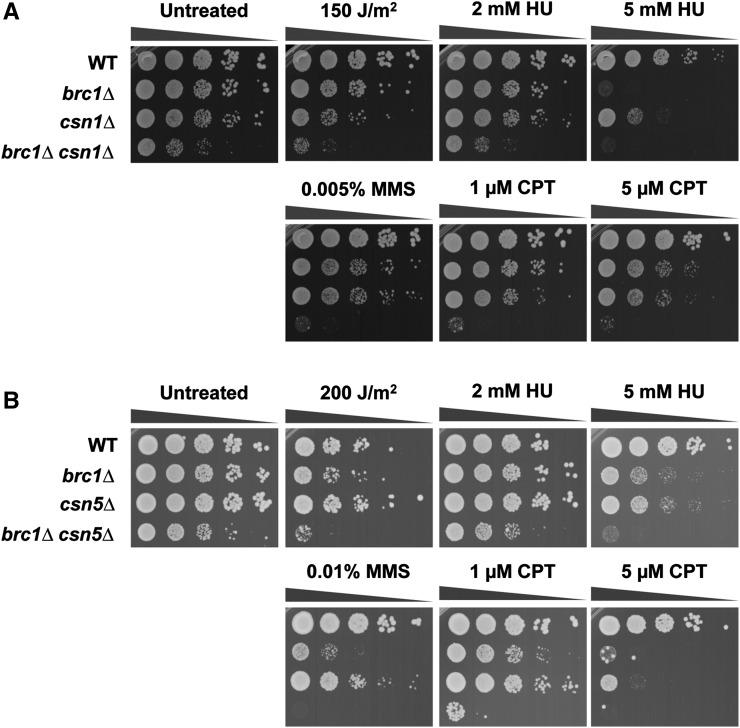
Critical requirement of COP9/Signalosome (CSN) in *brc1Δ* cells. Genetic interaction between Brc1 and Csn1 (A) or Csn5 (B). 10-fold serial dilutions of the indicated strains were exposed to the indicating DNA-damaging agents. Plates were incubated at 30° for 3−4 d. CPT, camptothecin; HU, hydroxyurea; MMS, methyl methanesulfonate; WT, wild type.

**Figure 2:**
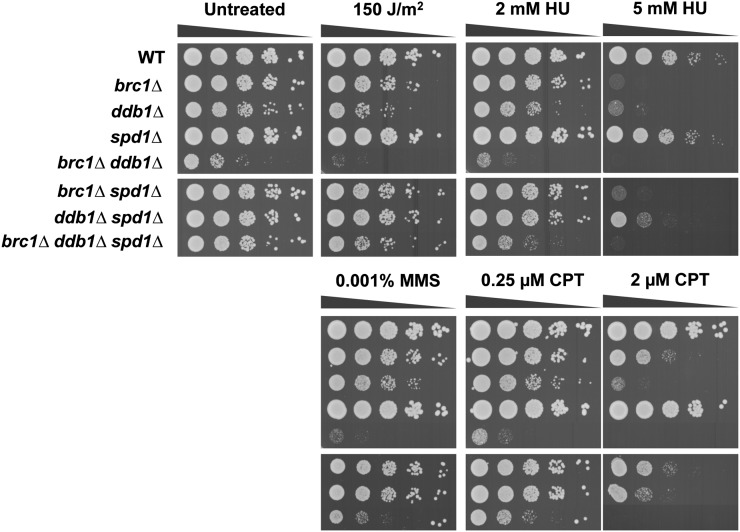
Genetic interactions among Brc1, Ddb1, and Spd1. 10-fold serial dilutions of the indicated strains were exposed to the indicating DNA damaging agents. Plates were incubated at 30° for 3−4 d. CPT, camptothecin; HU, hydroxyurea; MMS, methyl methanesulfonate; WT, wild type.

We sought to confirm and extend the analyses of a select group of the most interesting SSL interactions (Table 3). We created and tested new null alleles of COP9 signalosome complex genes (*csn1*, *csn5*), DNA repair and cell-cycle checkpoint genes (*ddb1*, *rad26*, *rad17*, *srs2*, *pnk1*), a DNA replication gene (*swi3*), and chromatin-silencing and remodeling genes (*sde2*, *raf1*, *set1*, *snt1*). As judged by colony size and density of double mutants compared with single mutants in serial dilution assays, we detected strong negative interactions with *csn1*, *ddb1*, and *pnk1*; moderate negative genetic interactions with *csn5*, *rad26*, *rad17*, *srs2*, *swi3*, and *sde2*; and only weak genetic interactions with *raf1* and *snt1*. In most cases these negative genetic interactions were strikingly enhanced when cells were exposed to low or moderate doses of genotoxins such as UV light, HU, CPT, or MMS (Figure 1, Figure 2, Figure S1, and Figure S2). Notably, the negative genetic interactions with *raf1* and *snt1* became obvious in the presence of these genotoxins. Of the 13 SSL interactions that were retested, only *set1* failed to confirm the results of the large-scale E-MAP screen (Figure S2C).

**Table 3:** Summary of genetic interactions involving *brc1Δ*

Allele	Function	Untreated	UV	HU	CPT	MMS
*csn1Δ*	Signalosome complex subunit	YES	YES	YES	YES	YES
*ddb1Δ*	Damage DNA binding protein Part of the ubiquitin ligase complex	YES	YES	YES	YES	YES
*csn5Δ*	Signalosome complex subunit	Yes	Yes	Yes	Yes	Yes
*rad26Δ*	Cell cycle arrest	Yes	YES	YES	YES	YES
*rad17Δ*	RFC related checkpoint protein	Yes	−	YES	YES	YES
*srs2Δ*	ATP-dependent DNA helicase	Yes	YES	YES	YES	YES
*pku80Δ*	Ku protein (NHEJ)	No	YES	YES	YES	YES
*pnk1Δ*	DNA kinase/phosphatase (SSBR)	YES	YES	YES	YES	YES
*swi3Δ*	Replication fork protection complex subunit	Yes	Yes	Yes	Yes	Yes
*sde2Δ*	Silencing defective protein	Yes	YES	YES	YES	YES
*raf1Δ*	Rik1-associated factor	No	Yes	Yes	Yes	Yes
*set1Δ*	Histone lysine methyltransferase	No	No	R	No	No
*snt1Δ*	Set3 complex subunit	No	No	Yes	Yes	Yes
*csi1Δ*	Chromosome segregation impaired protein 1	No	R	R	R	R
*msh2Δ*	MutS protein homolog 2	No	No	No	Yes	Yes
*cbp1Δ*	CENP-B homolog	No	No	No	Yes	Yes

Double mutants were assessed for growth in the absence or presence of specified genotoxins. UV, ultraviolet; HU, hydroxyurea; CPT, camptothecin; MMS, methyl methanesulfonate; YES, strong negative interaction; Yes, negative interaction; No, no genetic interaction; R, suppression.

We also retested the positive genetic interactions between *brc1* and *csi1* (centromere clustering protein), *msh2* (mismatch DNA repair MutS homolog), and *cbp1* (centromeric DNA binding protein CENP-B homolog). None of these interactions were confirmed in dilution assays performed in the absence of genotoxins; however, the *csi1Δ* allele clearly suppressed *brc1Δ* genotoxin sensitivity (Figure S3A). In contrast, double mutants involving *brc1Δ* and *msh2Δ* or *cbp1Δ* grew more poorly than single mutants when tested in the presence of genotoxins (Figure S3, B and C).

### Deneddylation-independent activities of the CSN are especially critical in the absence of Brc1

Brc1 displays negative genetic interactions with Csn1 and Csn5, which are members of the CSN. The hallmark activity of CSN consists of the deneddylation of the cullin subunit of cullin-RING E3 ligases (CRLs), which favors CRL disassembly to maintain cycles of CRL assembly and disassembly that are needed protect CRL components from self-destruction (Cope *et al.* 2002). As mentioned previously, we confirmed the strong negative genetic interaction between *brc1* and *csn1* by creating and testing a new *csn1Δ* null allele, which caused a modest growth defect that was substantially enhanced when combined with *brc1Δ* (Figure 1A, untreated). As previously described, the *csn1Δ* cells were mildly sensitive to the topoisomerase I inhibitor CPT, the DNA alkylating agent MMS, UV light, and the ribonucleotide reductase (RNR) inhibitor HU, which slows DNA replication (Hayles *et al.* 2013; Mundt *et al.* 1999). In comparison with *brc1Δ* or *csn1Δ* strains, the double mutant *brc1Δ csn1Δ* cells displayed very poor growth in the presence of these genotoxins (Figure 1A). We also confirmed the negative genetic interaction between *brc1* and *csn5* (Figure 1B, untreated). Although initial studies indicated that *csn5Δ* mutants did not share the genotoxin sensitivity phenotype of *csn1Δ* cells (Mundt *et al.* 2002), in our assays *csn5Δ* cells were sensitive to chronic exposure to HU, CPT and MMS, although less so than *csn1Δ* or *brc1Δ* mutants. Double mutant *brc1Δ csn5Δ* cells grew quite poorly in the presence of these genotoxins (Figure 1B).

Ddb1 is a core member of CLR4 (Cul4-Ddb1 RING ligase) that is target of CSN deneddylation activity. We confirmed that the modest growth defect caused by *ddb1Δ* was substantially enhanced when combined with *brc1Δ* (Figure 2 untreated). The *ddb1Δ* cells were mildly sensitive to UV, MMS, and HU (Zolezzi *et al.* 2002) and CPT (Figure 2). We found that double mutant *brc1Δ ddb1Δ* cells were highly sensitive to these DNA-damaging agents (Figure 2).

The negative genetic interactions between Brc1 and members of the CSN and CRL4 ubiquitin ligase imply that Brc1 and CSN independently act in genome maintenance pathways that are partially complementary. The JAMM motif within the MPN domain of Csn5 is responsible for the CRL deneddylation activity of the CSN (Cope *et al.* 2002; Mundt *et al.* 2002; Zhou *et al.* 2001). This catalytic function is dependent of the integrity of the complex (Mundt *et al.* 2002). The weaker SSL for the *brc1Δ csn5Δ* double mutant (E-MAP score = −3.8) compared with the *brc1Δ csn1Δ* double mutant (E-MAP score = −15.1) suggests that deneddylation-independent activities of the CSN are especially critical in the absence of Brc1.

### Increased RPA and Rad52 foci in *csn1Δ* cells

**Figure 3:**
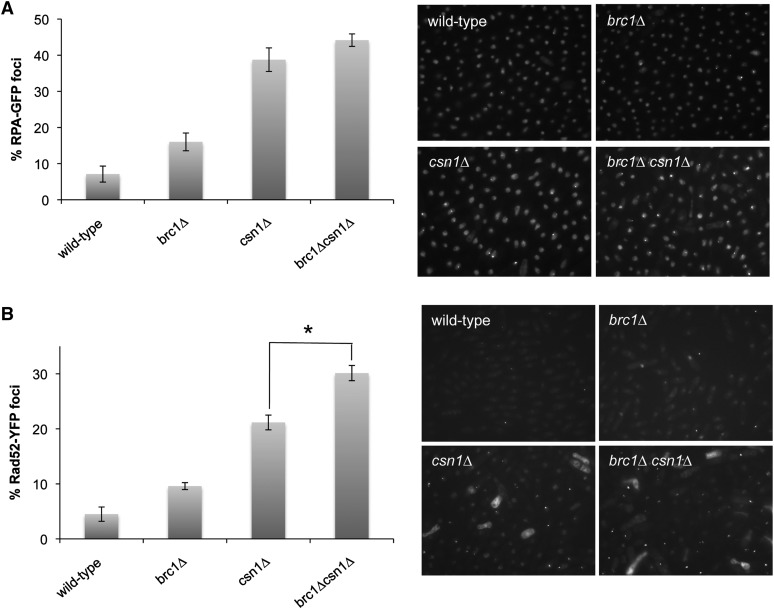
Increased Rad52 and RPA foci in *csn1Δ* cells. Cells expressing Rad52-yellow fluorescentprotein (A) or RPA(Ssb1)-green fluorescent protein (B) were cultured in minimal medium at 25° until mid-log phase. Foci were scored in three independent experiments. Rad52 foci in *brc1Δcsn1Δ* cells are statistically increased relative to *csn1∆* cells (two-tailed Student’s *t*-test, *P*-value 0.0015). Error bars correspond to standard deviations of the means. Asterisk depicts statistically significant differences between the bracketed strains as determined by a two-tailed Student’s *t*-test, *P*-value ≤ 0.05.

The SSL interaction between *csn1Δ* and *brc1Δ* suggested that the double mutant suffers increased rates of DNA damage or is unable to efficiently repair DNA lesions. To test this proposition we first monitored the formation of Replication Protein A (RPA) foci in *csn1Δ* and *brc1Δ csn1Δ* cells. RPA is the major single-strand DNA (ssDNA)-binding protein in eukaryotic cells (Parker *et al.* 1997). Formation of RPA foci in untreated cells is thought to arise predominantly from replication fork stalling or collapse and subsequent homology-directed repair that involve resection of DNA ends to generate 3′ ssDNA tails. For our assays we used strain in which Ssb1 (aka Rad11 in fission yeast), which is the largest subunit of RPA, was expressed with a YFP tag from the endogenous *ssb1*^+^ locus. In agreement with previous studies (Bass *et al.* 2012), we observed a significant increase in cells with RPA foci in the *brc1Δ* (16.0%) strain compared with wild type (7.1%). There was a much larger increase in cells with RPA foci in the *csn1Δ* strain (38.8%). There was a further small increase in the *brc1Δ csn1Δ* strain (44.2%) although the difference with *csn1Δ* was not quite statistically significant (p-value = 0.08) (Figure 3A). We also monitored foci formation of Rad52, previously known as Rad22, which is essential for all forms of homology-directed repair in fission yeast (Meister *et al.* 2003). As observed previously (Williams *et al.* 2010), the frequency of Rad52-YFP foci was significantly increased in *brc1Δ* cells (9.6%) as compared with the wild type. The incidence of cells with Rad52 foci was higher in the *csn1Δ* strain (21.2%), and there was a further significant increase in the *brc1Δ csn1Δ* strain (30.1%) (Figure 3B). These findings suggest that Brc1 prevents replication fork instability in CSN-defective cells.

### Defective relief of RNR inhibition in *csn1Δ* and *ddb1Δ* cells contributes to SSL interaction with *brc1Δ*

**Figure 4:**
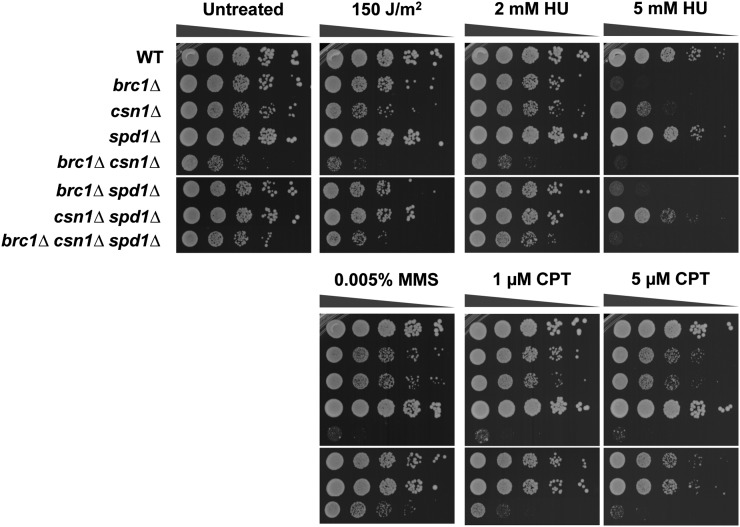
Genetic interactions among Brc1, Csn1, and Spd1. 10-fold serial dilutions of the indicated strains were exposed to the indicating DNA damaging agents. Plates were incubated at 30° for 3−4 d. CPT, camptothecin; HU, hydroxyurea; MMS, methyl methanesulfonate; WT, wild type.

Ddb1, Cullin 4 (Pcu4), and CSN subunits, Csn1 and Csn2, are required for degradation of Spd1, which is an inhibitor of RNR (Holmberg *et al.* 2005). As *spd1* deletion partially suppresses genotoxin sensitivity in *ddb1Δ* and *csn1Δ* cells, we investigated whether the defect in degrading Spd1 contributed to the SSL interaction between *brc1Δ* and *ddb1Δ* or *csn1Δ*. Our genetic analyses revealed that *spd1* deletion substantially suppressed the growth defects in *brc1Δ ddb1Δ* and *brc1Δ csn1Δ* backgrounds (Figure 2 and Figure 4, untreated). This suppression was also evident to varying degrees in cells treated with a panel of genotoxins (UV, HU, MMS, and CPT) (Figure 2 and Figure 4). Taken together, these data indicate that the defect in relieving Spd1-mediated inhibition of RNR in *ddb1Δ* and *csn1Δ* cells is a major factor in the SSL interactions with *brc1Δ*, although other pathways involving Csn1 and Ddb1 must also contribute to these negative genetic interactions.

### Requirement for γH2A in *csn1Δ* cells

**Figure 5:**
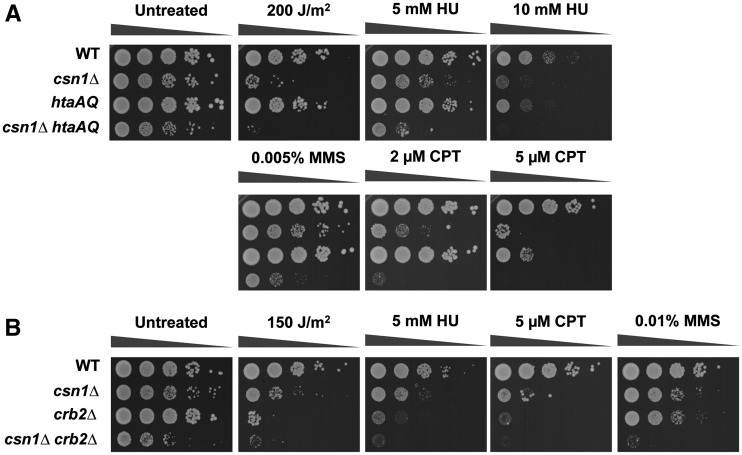
Critical requirement of γH2A in *csn1Δ* cells. Genetic interaction between Csn1 and *htaAQ* (A) or Crb2 (B). 10-fold serial dilutions of the indicated strains were exposed to the indicating DNA-damaging agents. Plates were incubated at 30° for 3−4 d. CPT, camptothecin; HU, hydroxyurea; MMS, methyl methanesulfonate; WT, wild type.

Rad3 checkpoint kinase, the fission yeast ortholog of mammalian ATR and budding yeast Mec1, plays a central role in replication stress response triggered by stalled and collapsed replication forks (Boddy *et al.* 1998; Lindsay *et al.* 1998). Rad3 has a number of important substrates, including the serine in the SQE motif in the C-terminal tail of histone H2A (Nakamura *et al.* 2004). Phospho-H2A, also known as γH2A, serves as a chromatin recruitment platform for Brc1, Crb2, and Mdb1, which all bind γH2A through their C-terminal BRCT domains. To assess whether γH2A is important in the absence of CSN complex, we constructed a *csn1Δ* strain in which both histone H2A genes contained a mutation that changed the C-terminal SQE phosphorylation motif to AQE (*hta1-S129A hta2-S128A*), which is the so-called *htaAQ* genotype. In comparison with the parental strains, the *csn1Δ htaAQ* strain displayed a reduced growth phenotype that was particularly evident in the presence of a panel of genotoxins (UV, HU, CPT, MMS) (Figure 5A).

Both Brc1 and Crb2 have well-established roles in DNA damage responses that are required for survival of genotoxic stress. Crb2 is required for activation of the checkpoint kinase Chk1 in response to DNA damage. As Chk1 was reported to have a synthetic growth defect with *csn1Δ*, we expected that Crb2 would be important in *csn1Δ* cells. Indeed, our studies revealed that *csn1Δ crb2Δ* cells grew poorly compared to the parental strains and this defect was accentuated in the presence of the panel of genotoxins (Figure 5B). These data suggest that γH2A interactions with both Brc1 and Crb2 are important in *csn1Δ* cells.

## Discussion

In this study we used E-MAP to explore the genetic interactions of *S. pombe* Brc1, a protein with six BRCT domains that binds γH2A and is important for survival of replication stress. Brc1 was identified as an allele-specific high-copy suppressor of *smc6-74* (Verkade *et al.* 1999), it becomes essential in strains with compromised Smc6 or Nse4 functions, and *brc1Δ* is also has strong negative genetic interactions with conditional alleles of *rad60* and *top2* (Morikawa *et al.* 2004; Pebernard *et al.* 2006; Verkade *et al.* 1999). Among the 56 SSL interactions revealed in our E-MAP analysis, only four were previously detected through classical genetic analyses: *apn2* (E-MAP score = −14.58), encoding an apurinic/apyrimidinic endonuclease and *rad2* (E-MAP score = −2.98), encoding a FEN1 endonuclease, both of which are involved in base excision repair (Alseth *et al.* 2004; Alseth *et al.* 2005); *mms22* (E-MAP score = −2.44), encoding a DNA repair protein that forms a complex with Mms1 (Dovey and Russell 2007); and *ssb3* (E-MAP score = −10.97), encoding the nonessential small subunit of the replication protein A (Cavero *et al.* 2010). An additional nine genes (*csn1*, *hrq1*, *pmt3*, *rad1*, *rad17*, *SPCC1442.02*, *srs2*, *pnk1*, and *csn5*) found in our E-MAP were detected in previous genetic interaction screens (Beltrao *et al.* 2009; Ryan *et al.* 2012). This list is strongly enriched for genes involved in genome stability but our results show that it is not exhaustive. Our screen revealed an additional 11 SSL interactions with genes that have established roles in genome protection, including *ddb1*, *rad9*, *pku80*, *hus1*, *rad26*, *dcd1*, *nse6*, *swi3*, *csn71*, *dbl1*, and *mrc1*. Indeed, GO analysis of the SSL interactions identified in our screen revealed very strong enrichment for genes involved in DNA repair and checkpoint functions.

Some of the new SSL interactions detected in our screen validated previous findings. For example, the SSL interactions with the Rad1 subunit of the Rad9-Hus1-Rad1 checkpoint clamp and the Rad17 subunit of the Rad17-RFC checkpoint clamp loader were uncovered in previous screens (Beltrao *et al.* 2009; Ryan *et al.* 2012). Our screen additionally detected SSL interactions with Rad9 and Hus1, thereby detecting all four genes in the Rad9, Hus1, Rad1, and Rad17 genetic epistasis group. Indeed, it is impressive that E-MAP scores for *rad1*, *rad9*, and *rad17* were so similar, −8.7, −8.7, and −8.4, respectively, which attests to high accuracy of the measurements in this particular implementation of the E-MAP procedure.

Similarly, previous *brc1Δ* E-MAP screens identified *csn1* and *csn5* (Beltrao *et al.* 2009; Ryan *et al.* 2012), which are subunits of the CSN complex required for cullin deneddylation (Mundt *et al.* 2002), whereas our screen identified these genes as well as the CSN subunit *csn71*. In addition, our *brc1Δ* E-MAP screen detected an SSL interaction with Ddb1, which is required for degradation of the RNR inhibitor Spd1 (Bondar *et al.* 2004; Holmberg *et al.* 2005).

Among the CSN subunits identified in our screen, we found that negative genetic interaction was strongest with *csn1*. This observation suggests that loss of the deneddylation activity dependent on the Csn5 subunit is not fully responsible of the SSL interaction between Brc1 and CSN, nor are the SSL interactions totally explained by the role of Ddb1/Csn1 in controlling RNR activation through Spd1 degradation. These results suggest that CSN and Brc1 function in parallel in response to DNA damage and contribute to genome stability through multiple pathways. Supporting this idea, our studies revealed that *csn1Δ* mutants have increased numbers of RPA and Rad52 foci. Our studies also reveal the importance of γH2A in the absence of CSN, with our data indicating that binding of both Brc1 and Crb2 to γH2A is important in response to replication stress. Interestingly, deregulation of CSN and its interactions are related to multiple cancers, making CSN an interesting target for cancer therapy (Fuzesi-Levi *et al.* 2014; Lee *et al.* 2011; Richardson and Zundel 2005).

The SSL interaction of *brc1* with *nse6* provides clues about the functional relationships between Brc1 and the Smc5−Smc6 complex. As mentioned previously, Brc1 was initially discovered as an allele-specific, high-copy suppressor of *smc6-74* (Verkade *et al.* 1999). This type of genetic interaction often indicates a physical association; for example, the missense mutation in *smc6-74* might impair binding to Brc1, which is a defect that might be suppressed by increasing the total cellular concentration of Brc1. It is unknown if Brc1 associates with the Smc5−Smc6 holocomplex; however, the Brc1 homolog in *S. cerevisiae* coprecipitates with multiple subunits of the Smc5−Smc6 holocomplex (Ohouo *et al.* 2010). Nse5 and Nse6 form a heterodimer that is part of the Smc5-Smc6 holocomplex and is required for many or all of its DNA repair functions, but unlike other subunits of the holocomplex Nse5 and Nse6 are not essential for cell viability (Pebernard *et al.* 2006). The SSL interaction of *brc1* with *nse6* detected in our screen strongly indicates that the DNA repair functions of Brc1 and Smc5-Smc6 holocomplex are at least partially independent.

The SSL interaction of *brc1* with *mrc1* is novel and provides insights about the requirement for Brc1 in the response to replication stress. Mrc1 (mediator of replication checkpoint) was discovered by screening for mutations that cause hydroxyurea sensitivity and are rescued by overproduction of the replication checkpoint kinase Cds1/Chk2 (Tanaka and Russell 2001). Mrc1 is conserved in budding yeast and mammals in which it is known as Mrc1 and claspin, respectively (Alcasabas *et al.* 2001; Kumagai and Dunphy 2000). The *mrc1*^+^ gene in fission yeast is periodically transcribed during S-phase in the cell cycle and recruiting Cds1 to stalled replication forks by Mrc1 is required for its efficient activation of Cds1. Mrc1 appears to have both Rad53-dependent and -independent functions that stabilize replication forks in *S. cerevisiae* (Katou *et al.* 2003; Osborn and Elledge 2003), but it is unclear whether Mrc1 has Cds1-independent activities in fission yeast (Nitani *et al.* 2006). In our *brc1Δ* E-MAP we uncovered a significant SSL interaction with *mrc1Δ* (E-MAP = −2.52) but not with *cds1Δ* (E-MAP = −0.60), even though *cds1Δ* mutants are more severely sensitive to HU (Tanaka and Russell 2001). These data indicate that the absence of Brc1 enhances the requirement for a Cds1-independent function of Mrc1 in stabilizing replication forks.

Similar conclusions are suggested by the SSL interaction of *brc1Δ* with *swi3Δ* (E-MAP = −4.6). Swi3 binds Swi1 to form the fork protection complex that stabilizes stalled replication forks (Noguchi *et al.* 2003; Noguchi *et al.* 2004). This activity is required for robust activation of Cds1 in response to HU treatment and other forms of replication stress. However, the absence of an SSL interaction between *brc1Δ* and *cds1Δ* mutations suggests that Cds1-independent activity of Swi1-Swi3 fork protection complex is especially critical in the absence of Brc1.

Although we focused on the SSL interactions identified in our *brc1Δ* E-MAP, we did confirm the alleviating (positive) interaction with *csi1* (E-MAP = +2.14). Csi1 was implicated in centromere clustering during interphase through its interaction with Sad1 in the spindle pole body and it also has a role in tethering spindle-stabilizing factors to the spindle pole body for promoting bipolar spindle assembly (Hou *et al.* 2012; Zheng *et al.* 2014). The involvement of Csi1 in these processes is interesting in light of our evidence that recruiting Brc1 to γH2A in pericentromeric heterochromatin during S-phase contributes to maintaining the heterochromatic state, which is required for efficient chromosome segregation during nuclear division (Lee *et al.* 2013). Indeed, genetic assays indicate that Brc1 is required for mitotic chromosome stability, which suggests a role for Brc1 in chromosome segregation (Verkade *et al.* 1999). Furthermore, we found that *brc1Δ* cells are moderately sensitive to the microtubule-destabilizing drug thiabendazole and display increased rates of chromosome missegregation in the presence of thiabendazole (Lee *et al.* 2013). These effects of Brc1 correlate with the genetic data linking Brc1 to the Smc5-Smc6 complex (Verkade *et al.* 1999) and data showing that the holocomplex localizes around centromeres during S-phase and defects in the complex increase the frequency of lagging chromosomes during nuclear division (Pebernard *et al.* 2008). However, despite these striking correlations, it is unclear why a defect in Csi1 function should alleviate the requirement for Brc1 as suggested by our genetic suppression data. In this regard it is interesting that *csi1Δ* cells are sensitive to the DNA-damaging agent 4-nitroquinoline 1-oxide, which causes replication stress by producing bulky adducts in DNA (Deshpande *et al.* 2009). Our studies further indicated that *csi1Δ* cells are mildly sensitive to UV light, HU, and CPT (Figure S3). Most strikingly, the *csi1Δ* mutation effectively suppresses sensitivity of *brc1Δ* cells to these genotoxins and MMS. Again, it is unobvious how this suppression happens, although we note that there are some well-known examples of mutations in different DNA repair pathways having suppressive effects; for example, eliminating the Ku complex required for nonhomologous end joining (NHEJ) suppresses defects in the Mre11-Rad50-Nbs1 complex and Ctp1 that are required for homologous recombination repair (Langerak *et al.* 2011), and the *rad51Δ* mutations suppresses UV sensitivity of *nse6Δ* mutants (Pebernard *et al.* 2006).

Finally, the list of SSL interactions derived from the E-MAP studies for Brc1 (56 SSL interactions) and Rtt107 (33 SSL interactions) reveal remarkably little overlap, with only one gene, the ATP-dependent DNA helicase *srs2/SRS2*, being found in both screens. In *S. pombe*, deletion of *srs2* causes elevated rates of spontaneous recombination (Doe and Whitby 2004). Furthermore, deletion of *brc1* suppressed the hyper-recombination phenotype of an *srs2Δ* strain (Bass *et al.* 2012). This small degree of overlap suggests major functional differences between Brc1 and Rtt107 despite their similarities in domain organization and a shared mechanism of localizing to DNA lesions through C-terminal BRCT domains binding γH2A (Li *et al.* 2012; Williams *et al.* 2010). However, comparing all genetic interactions identified by classical genetic analyses and E-MAP suggests an additional degree of overlap for Brc1 and Rtt107. For example, classical genetic interactions uncovered strong negative interactions with Rqh1 and Sgs1, which are orthologous DNA helicases of the RecQ family. Nevertheless, the unexpectedly low overlap for both E-MAP lists and GO process terms suggests significant functional differences between Brc1 and Rtt107, reflecting the large evolutionary divergence between *S. pombe* and *S. cerevisiae*.
